# Non-Myelofibrosis Chronic Myeloproliferative Neoplasm Patients Show Better Seroconversion Rates after SARS-CoV-2 Vaccination Compared to Other Hematologic Diseases: A Multicentric Prospective Study of KroHem

**DOI:** 10.3390/biomedicines10112892

**Published:** 2022-11-11

**Authors:** Zrinka Sertić, Marko Lucijanić, Sandra Bašić-Kinda, Ranka Serventi Seiwerth, Vlatka Periša, Dubravka Sertić, Božena Coha, Dražen Pulanić, Zinaida Perić, Lana Desnica, Mirta Mikulić, Marijo Vodanović, Ivo Radman-Livaja, Dragana Šegulja, Dunja Rogić, Toni Valković, Igor Aurer, Nadira Duraković

**Affiliations:** 1 Institute for Emergency Medicine Krapina-Zagorje County, 49 000 Krapina, Croatia; 2 School of Medicine, University of Zagreb, 10 000 Zagreb, Croatia; 3 Clinical Hospital Dubrava, 10 000 Zagreb, Croatia; 4 University Hospital Centre Zagreb, 10 000 Zagreb, Croatia; 5 University Hospital Centre Osijek, 31 000 Osijek, Croatia; 6 Faculty of Medicine Osijek, University J.J. Strossmayer Osijek, 31 000 Osijek, Croatia; 7 General Hospital “Dr. Josip Benčević” Slavonski Brod, 35 000 Slavonski Brod, Croatia; 8 University Hospital Center Rijeka, 51 000 Rijeka, Croatia; 9 Medical School, University of Rijeka, 51 000 Rijeka, Croatia

**Keywords:** SARS-CoV-2 vaccination, COVID-19, chronic myeloproliferative neoplasms, chronic myeloid leukemia, non-Hodgkin’s lymphoma, chronic lymphocytic leukemia, humoral immune response, anti-CD20 monoclonal antibody

## Abstract

Disease- and treatment-mediated immunodeficiency might render SARS-CoV-2 vaccines less effective in patients with hematologic diseases. We performed a prospective non-interventional study to evaluate humoral response after one and two doses of mRNA-1273, BNT162b2, or ChAdOx1 nCoV-19 vaccine in 118 patients with different malignant or non-malignant hematologic diseases from three Croatian treatment centers. An electrochemiluminescent assay was used to measure total anti-SARS-CoV-2 S-RBD antibody titers. After one vaccine dose, 20/66 (33%) achieved seropositivity with a median antibody titer of 6.1 U/mL. The response rate (58/90, 64.4%) and median antibody titer (>250 U/mL) were higher after two doses. Seropositivity varied with diagnosis (overall *p* < 0.001), with the lowest rates in lymphoma (34.6%) and chronic lymphocytic leukemia (52.5%). The overall response rate in chronic myeloproliferative neoplasms (CMPN) was 81.3% but reached 100% in chronic myeloid leukemia and other non-myelofibrosis CMPN. At univariable analysis, age > 67 years, non-Hodgkin’s lymphoma, active treatment, and anti-CD20 monoclonal antibody therapy increased the likelihood of no vaccine response, while hematopoietic stem cell recipients were more likely to respond. Age and anti-CD20 monoclonal antibody therapy remained associated with no response in a multivariable model. Patients with the hematologic disease have attenuated responses to SARS-CoV-2 vaccines, and significant variations in different disease subgroups warrant an individualized approach.

## 1. Introduction

In a time span of months from its first isolation as the cause of a local respiratory disease outbreak, novel coronavirus (SARS-CoV-2) became the leading cause of death from infectious disease worldwide. Initial reports demonstrated several-fold higher overall mortality in patients with hematologic malignancy compared to the general population in the same pandemic period [1,2,3,4]. While mortality varied between different hematologic conditions and was highest in patients with acute leukemia and bone marrow failure, it still exceeded 30% in every disease group [1]. 

With a prompt response from the scientific community, landmark phase III randomized controlled trials (RCTs) administering two doses of SARS-CoV-2 vaccines demonstrated over 90% efficacy in preventing symptomatic coronavirus disease (COVID-19) [5,6,7]. Following authorization from the European Medicines Agency, the BNT162b2 mRNA (BioNTech, Mainz, Germany/Pfizer, New York, NY, USA) became available in Croatia in late December 2020, followed by ChAdOx1 nCoV-19 (AstraZeneca, Cambridge, UK) and mRNA-1273 (Moderna, Cambridge, MA, USA) in January 2021. Patients with hematologic conditions were among those prioritized for early vaccination. However, as initial RCTs excluded immunocompromised populations, it was unknown how their vaccine response would compare to immunocompetent trial subjects. Hematologic malignancy is already associated with an attenuated response to other vaccines [8,9,10], and those who had received B-cell-depleting therapy had no antibody response with prolonged viral shedding following SARS-CoV-2 infection [11]. Several single-center studies soon raised concern about suboptimal seroconversion rates following a standard two-dose regimen of SARS-CoV-2 vaccines [12,13,14,15].

Immune response in patients with hematologic disorders depends on disease-specific immunologic environments and targeted therapeutic modalities. Identifying which subgroups of these high-risk patients might not respond to classic vaccination schemes would influence clinical decision-making as they could be candidates for alternative interventions. We aimed to examine quantitative and qualitative humoral vaccine response in a prospectively enrolled cohort with different malignant and non-malignant hematologic diseases in a multicentric setting. An additional explorative analysis was performed to identify potential predictors of no humoral response after two vaccine doses. The study was conducted by the Croatian Cooperative Group for Hematologic Diseases (KroHem).

## 2. Materials and Methods

Between January and July 2021, we prospectively enrolled patients from University Hospital Centre Zagreb, University Hospital Centre Osijek, and General Hospital “Dr. Josip Benčević” Slavonski Brod. All patients were adults (≥18 years) with a history of the malignant or non-malignant hematologic disease who had received at least one dose of either mRNA-1273 (Moderna, Cambridge, MA, USA), BNT162b2 mRNA (BioNTech, Mainz, Germany/Pfizer, New York, NY, USA), or ChAdOx1 nCoV-19 (AstraZeneca, Cambridge, UK) COVID-19 vaccine. The exclusion criterion was previous COVID-19 infection. Patients followed the recommended dosing intervals: 21 days for BNT162b2, 28 days for mRNA-1273, and 12 weeks for ChAdOx1 nCoV-19. The response was evaluated at least 7 days following the last vaccine dose. The study was approved by hospital ethics committees, and all participants provided written informed consent.

Seroconversion after vaccination was determined using a serological immunoassay registered for quantitative measurement of antibodies against the SARS-CoV-2 spike protein receptor-binding domain (RBD). This assay, Elecsys Anti-SARS-CoV-2 S (Roche Diagnostics, Mannheim, Germany), measures total antibodies against SARS-CoV-2 S glycoprotein by using SARS-CoV-2 S RBD recombinant antigens that predominantly capture anti-SARS-CoV-2 S immunoglobulin G (IgG), but also IgA and IgM [16]. 

In order to identify the previous infection, anti-SARS-CoV-2 nucleocapsid antigen antibodies (including IgG) were qualitatively assessed. The used reagent, Elecsys Anti-SARS-CoV-2 (Roche Diagnostics, Mannheim, Germany), consists of a recombinant protein representing the N antigen in a double-antigen sandwich assay format.

Both assays were performed by Cobas e801 analyzer (Roche Diagnostics, Mannheim, Germany) according to manufacturer instructions and by following principles. Biotinylated and ruthenylated antigens, in the presence of corresponding antibodies, create double-antigen sandwich immune complexes. The complexes bind to the solid phase by an interaction between biotin and streptavidin after the addition of streptavidin-coated microparticles. Microparticles are magnetically captured to the electrode surface in the measuring cell. Electrochemiluminescence is then induced by applying a voltage and measured with a photomultiplier. The signal yield increases with the antibody titer. A positive response was defined as >0.8 U/mL with lower and upper limits of quantification of 0.4 U/mL and 250 U/mL, respectively [16]. These numerical results in U/mL are equivalent to the 1st WHO International Standard for anti-SARS-CoV 2 immunoglobulin BAU/mL [17].

We reviewed in-hospital electronic records for demographic and clinical characteristics, including underlying hematologic disease, date of diagnosis, current and previously received treatment, the number of received treatment lines, application of anti-CD20 monoclonal antibodies (mAbs) or corticosteroid therapy in six months before vaccination, hematopoietic stem cell transplantation (HSCT) and total serum IgG levels prior to vaccination. Participants were followed until December 2021 for outcomes that included symptomatic SARS-CoV-2 infection, severe forms of COVID-19 requiring oxygen supplementation or ICU admission, and death.

Categorical variables were summarized with counts and frequencies, and continuous variables with medians and interquartile ranges. Response after the first versus second dose was compared with Wilcoxon sign-rank test for antibody titers and McNemar’s χ^2^ test for seropositivity rates. Subgroup comparisons for those who received two doses were performed with Mann-Whitney U, χ^2^, or Fisher’s exact test as appropriate. ROC curve analysis was used to find optimized cut-off values of numerical variables regarding response to the second dose. Zou’s modified Poisson regression was performed to compute risk ratios (RRs) for no humoral response after two vaccine doses [18]. Variables of interest for regression analysis were: age > 67 years, sex, time from the second dose to antibody assessment, vaccine type, diagnosis of Non-Hodgkin’s lymphoma (NHL), chronic lymphocytic leukemia (CLL), multiple myeloma/amyloidosis (MM), acute leukemia (AL) or chronic myeloproliferative neoplasms (CMPN) including chronic myeloid leukemia (CML), polycythemia vera (PV), chronic eosinophilic leukemia or myelofibrosis (MF), time from diagnosis to the second dose, low total serum IgG, active treatment, any prior therapy, line of therapy, HSCT, HSCT > 1 year prior to vaccination, antiCD20 mAb therapy, corticosteroid therapy six months prior to vaccination and prednisone equivalent dose > 120 mg. Statistically significant variables at univariable analysis were included in a multivariable model. The final model was constructed with backward elimination. 

Statistical significance was determined at an α level of 0.05 throughout the analysis. All *p* values are based on two-sided tests. MedCalc statistical software (version 20.008, MedCalc Software Ltd., Ostend, Belgium) was used for inter-group comparisons; regression analysis and data visualization were performed with RStudio for OS X (version 1.2.1335, RStudio, PBC, Boston, MA, USA).

## 3. Results

We initially enrolled 141 participants. After excluding 23 patients for prior SARS-CoV-2 infection, as evidenced by the presence of anti-nucleocapsid antigen antibodies, 118 remained who had received at least one dose. Of those, 55.9% were male, with a median age of 65.2 years (IQR 52.3–71.9). The majority had received the Pfizer-BioNTech vaccine (66.9%), followed by Oxford-AstraZeneca (23.7%) and Moderna (9.3%). Lymphoid conditions were most prevalent with NHL in 32 participants (27.1%), CLL in 28 (23.7%), and Hodgkin lymphoma (HL) in three (2.5%), followed by MM in 17 (14.4%), AL in 15 (12.7%), CML in seven (5.9%), other CMPNs in nine (7.6%), myelodysplastic syndrome (MDS) in four (3.4%), aplastic anemia (AA) in two (1.7%) and immune thrombocytopenia (ITP) in one (0.8%). More than half were in active therapy at the time of vaccination (66.9%), while only 5.9% had not received any previous therapy. 

Data on the humoral response after the first dose were available in 66 patients. Median time from vaccination to evaluation after the first dose was 20 days (IQR 15–34). Twenty participants (30.3%) achieved a positive response with a median specific antibody titer of 6.1 U/mL. After receiving the first dose, two patients died from their primary disease, two received HSCT and were not eligible to receive a second dose, and two contracted COVID-19. Data after the second dose were not available for 22 patients. Of the 90 patients who had evaluable responses following a second dose, 58 (64.4%) achieved seropositivity with median specific antibody levels at the upper limit of quantification (250 U/mL). All first-dose responders maintained seropositivity after the second dose. In a subgroup analysis of those who provided samples after both doses (*n* = 38), the second dose significantly improved both the response rate (*p* = 0.041) and specific antibody titer (*p* < 0.001). 

**Figure 1:**
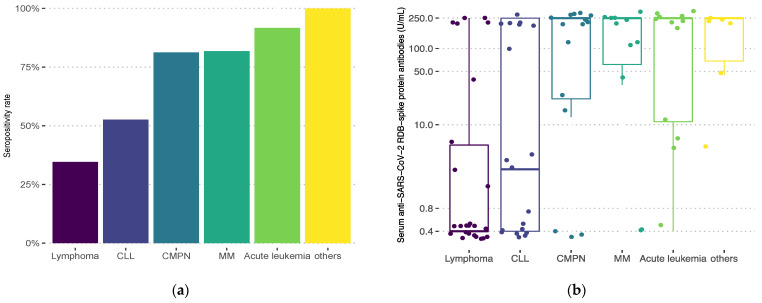
Seropositivity rates (**a**) and anti-SARS-CoV-2 spike protein receptor-binding domain antibody titers (**b**) after two vaccine doses according to diagnosis: lymphoma (Hodgkin’s or non-Hodgkin’s lymphoma; *n* = 26), chronic lymphocytic leukemia (CLL; *n* = 19), chronic myeloproliferative neoplasms (CMPN; chronic myeloid leukemia, polycythemia vera, myelofibrosis, chronic eosinophilic leukemia, *n* = 16), multiple myeloma/amyloidosis (MM; *n* = 11), acute leukemia (*n* = 12), others (myelodysplastic syndrome, aplastic anemia, idiopathic thrombocytopenic purpura, *n* = 6). Overall *p* < 0.001. Positive results are defined as >0.8 U/mL.

Seropositivity rates after two doses differed significantly with diagnosis (Figure 1, overall *p* < 0.001). Lymphoma patients had the lowest response rate (34.6%), followed by CLL (52.5%). Patients with CMPNs achieved an overall seropositivity rate of 81.3%. Response in the CML subgroup was 100% after two vaccine doses. Patients with PV and chronic eosinophilic leukemia all achieved response as well. Seronegative patients with CMPNs (*n* = 3) all had secondary MF and were on ruxolitinib therapy at the time of vaccination. Seropositive patients (*n* = 13) received either tyrosine-kinase inhibitors (TKIs), and hydroxyurea or had no specific chemoimmunotherapy. Antibody titers significantly varied with diagnosis as well (overall *p* < 0.001): lowest median titer levels were observed in lymphoma patients (0.4 U/mL, IQR 0.4–5.69) and those with CLL (2.6 U/mL, IQR 0.4–250), while other subgroups had median titers at the upper limit (Figure 1).

Characteristics of responders and non-responders after two vaccine doses are summarized in Table 1. At univariable analysis, age > 67 years, a diagnosis of NHL, active treatment, and anti-CD20 mAb therapy 6 months prior to vaccination increased the likelihood of no humoral response. HSCT overall and HSCT > 1 year prior to vaccination were associated with a significantly decreased risk of no response (Table 2). While the diagnosis of CMPN (RR 0.48, 95% CI 0.17–1.38; *p* = 0.172) did not reach statistical significance, as seroconversion of CML patients was 100%, risk could not be computed for this subgroup alone. CLL patients were at increased risk of no response (RR 1.46, 95% CI 0.82–2.61; *p* = 0.2). Incidentally, participants with AL (RR 0.21, 95% CI 0.03–1.40; *p* = 0.106) and MM (RR 0.46, 95% CI 0.13–1.66; *p* = 0.237) were more likely to respond, although none reached statistical significance. Any corticosteroid therapy or >120 mg equivalent prednisone dose did not significantly influence response, nor did vaccine type. In a multivariable model, only age > 67 years and anti-CD20 mAb therapy remained significantly associated with a lack of response (Table 2).

After a median follow-up of 8.2 months (IQR 7.5–8.8), five (4.2%) patients tested positive for SARS-CoV-2 after vaccination, of which two had received only one dose, and three had no humoral response despite receiving two doses. Two patients required in-hospital treatment and oxygen supplementation, and two died of COVID-19.

## 4. Discussion

Although a second vaccine dose was associated with a significantly better humoral response, the overall seroconversion rate of 64.4% in our cohort of hematologic patients was much lower than those reported in phase I/II RCTs, where virtually all participants seroconverted [19,20,21]. We observed the lowest seroconversion rates and antibody titers in patients with lymphoid malignancies, with only half of CLL and a third of lymphoma patients reaching the threshold for a positive response. On the other hand, overall seropositivity in CMPNs was 81.3%, and rates observed in CML and non-myelofibrosis CMPN patients were comparable to healthy trial subjects.

All chronic myeloproliferative disorders are associated with innate immune inhibition. However, CML patients in major and deep molecular responses seem to regain immunocompetency irrespective of TKI therapy or treatment-free remission [22]. Adequate disease control typically prevails in current-day CML cohorts, resulting in COVID-19 mortality similar to that of the general population in the same era of the pandemic [23], consistent reports of high seroconversion rates [12,14], and production of both humoral and cellular response to SARS-CoV-2 vaccines [24]. In contrast, BCR-ABL-1 negative CMPNs are associated with an increased risk of severe forms of COVID-19, most prominently MF, where case-fatality risk is 48% [25,26]. In our CMPN subgroup, those who failed to respond were all secondary MF patients on active treatment with ruxolitinib. Ruxolitinib was consistently associated with no humoral vaccine response [12,27,28,29], raising the question of treatment cessation in select high-risk patients during a standard SARS-CoV-2 vaccination schedule. In theory, immunomodulation could even be beneficial during the immune system hyperactivation phase of COVID-19, and discontinuation of ruxolitinib in active COVID-19 infection was associated with excess mortality [25]. However, Caocci et al. reported similar seroconversion rates in MF patients treated with ruxolitinib compared to other regimens [30], and discerning JAK1/2 inhibitor action from the effect of immune phenotypes in BCR-ABL1 negative CMPNs is difficult as both are known to cause deep immune system dysregulation [31,32]. Sudden ruxolitinib suspension is associated with severe inflammatory hyper reactions [33], and a phase III RCT of ruxolitinib plus standard of care versus placebo plus standard of care did not meet the composite endpoint of death, respiratory failure, or ICU admission in patients hospitalized for COVID-19 [34]. As the sum of current findings shows no clear benefit of withholding ruxolitinib in light of either SARS-CoV.2 vaccination or infection, the present agreement is to continue all active treatment in CMPNs [35].

To the extent of our knowledge, we report the lowest response rate (34.6%) in lymphoma patients to date. They were predominantly NHL patients, shown to have worse responses compared to HL [36], with a high proportion of those actively or recently treated with B-cell depleting agents. Comparably, Ghione et al. recorded a positive response in 36.3% of B-cell lymphoma patients, of whom almost a third received B-cell-directed treatment nine months before vaccination [37]. We observed a biologically plausible total failure of B-cell activation with median antibody titers below the lower limit of quantification in almost all participants who had received anti-CD20 mAbs. Surprisingly, the diagnosis of MM was associated with a tendency towards seropositivity. Previously reported seropositivity rates in MM patients widely varied (66–84%) [12,13,38,39], and so did their antibody titer range. In many seropositive patients with MM, antibody titers do not reach the upper limit of quantification even after two vaccine doses [39], revealing a more pronounced difference in immune response than observed by qualitative measurement alone.

In early reports of humoral response after two doses of the SARS-CoV-2 vaccine in HSCT recipients, 78% had quantifiable IgG (S-RBD) [40], and subsequent larger cohorts from Spanish and French registries reported similarly favorable seroconversion rates overall and up to 85% after autologous HSCT [41,42]. Of note, approximately 60% of HSCT recipients had antibody levels in a range likely to neutralize the virus [40,41]. In our study, prior HSCT was associated with a positive vaccine response. By withholding vaccination for the first three months, which is according to national guidelines, the majority produced detectable antibodies. Seroconversion rates in previous studies increased with time after transplantation. However, factors beyond the scope of our studies, such as lymphopenia, active GVHD, or immunosuppression, were independently predictive of response [40,41,42]. We propose that successful immune reconstitution after HSCT, disease remission, and no active chemoimmunotherapy, contributed to the result we observed, a similar pattern to that of the Lithuanian national cohort [27]. A confounding effect may also explain the high response rates observed in the AL subgroup since AL was the most common indication for HSCT. 

The study period coincided with the third pandemic wave in Croatia and the rise of the SARS-CoV-2 B.1.1.7 (Alpha) variant for which attenuated vaccine efficacy was observed, a continuing trend for variants of concern that followed [43,44,45]. Compared to healthy controls, hematologic patients showed an even faster decline in neutralizing antibody titers for B.1.351 (Beta) and B.167.2 (Delta) variants [46]. While routine serological response monitoring was not implemented even in high-risk populations, EMA and ECDC recommend administering a booster dose in all adults and, as of recently, even a second booster in the elderly and those with predisposing conditions. Administering a three-dose vaccine regimen was associated with lower hospitalization rates in the immunocompromised [47]. In hematologic patients, the additional dose produces a more robust humoral response in responders, although a fraction of non-responders seems to remain non-responsive [48,49,50]. Monin et al. observed a higher proportion of cellular versus humoral response in hematologic patients [51]. Subgroups with low seroconversion rates, such as rituximab-treated patients and those with lymphoid neoplasms, might still have some degree of SARS-CoV-2 vaccine protection from an independently induced cellular vaccine response [52,53,54].

Although most anti-RBD antibodies share neutralizing potential [55], without neutralization assays, we cannot definitively confirm the viral neutralization capacity of those who had seroconverted. A relatively small cohort, no control group, and real-world convenience sampling limit the interpretation of our results, and multiple treatment modalities in disease subgroups with few participants inevitably yielded residual confounding on the effect of disease and treatment. 

In conclusion, the original two-dose regimen of SARS-CoV-2 vaccines produces lower seropositivity rates overall in patients with hematologic disease. Response rates range significantly across different disease subgroups, from severely attenuated in lymphoid neoplasms to almost universal in BCR-ABL1-positive and non-myelofibrosis BCR-ABL1 negative CMPNs. Seroconversion rates after SARS-CoV-2 vaccination and the incidence of breakthrough infection in patients with hematologic disease should be investigated further in an interventional study design for which we stress the need for single-disease groups. 

## Figures and Tables

**Table 1:** Patient characteristics with regards to humoral response after two vaccine doses, defined as > 0.8 U/mL of serum anti-SARS-CoV-2 spike protein receptor-binding domain antibodies.

Variable	All Patients (*N* = 90)	Non-Responders (*n* = 32)	Responders (*n* = 58)	*p* Value
Age (years), median (IQR)	64.4 (53.5–71.5)	71 (66.3–75.9)	59.7 (49–66.8)	**<0.001**
Age > 67 years, No. (%)	35 (38.9%)	23 (71.9%)	12 (20.7%)	**<0.001**
Sex (female), No. (%)	41 (45.6%)	17 (53.1%)	24 (41.4%)	0.284
Time from second dose to antibody testing (days), median (IQR)	19 (14–27)	19 (13–26)	19 (14–27)	0.923
Vaccine type, No. (%)				overall *p* value 0.412
BNT162b2 (Pfizer-BioNTech)	65 (72.2%)	21 (65.6%)	44 (75.9%)	
ChAdOx1 nCoV-19 (Oxford-AstraZeneca)	16 (17.8%)	8 (25%)	8 (13.8%)	
mRNA-1273 (Moderna)	9 (10%)	3 (9.4%)	6 (10.3%)	
Diagnosis, No. (%)				overall * p * value **0.002**
Non-Hodgkin’s lymphoma	24 (26.7%)	17 (53.1%)	7 (12.1%)	** <0.001 **
Chronic lymphocytic leukemia	19 (21.1%)	9 (28.1%)	10 (17.2%)	0.226
Acute leukemia	12 (13.3%)	1 (3.1%)	11 (19.0%)	0.050
Chronic myeloid leukemia	7 (7.8%)	0	7 (12.1%)	** 0.042 **
Other chronic myeloproliferative neoplasms	9 (10%)	3 (9.4%)	6 (10.3%)	0.883
Multiple myeloma/amyloidosis	11 (12.2%)	2 (6.3%)	9 (15.5%)	0.199
Hodgkin’s lymphoma	2 (2.2%)	0	2 (3.4%)	0.288
Myelodysplastic syndrome	3 (3.3%)	0	3 (5.2%)	0.191
Non-malignant disorders °	3 (3.3%)	0	3 (5.2%)	0.191
Time from diagnosis to second dose (months), median (IQR) *	36.6 (17.4–82.9)	39.0 (11.4–73.8)	35.8 (18.5–86.9)	0.448
Total serum IgG < 7.0 g/L, No. (%) ^+^	19 (29.7%)	8 (33.3%)	11 (27.5%)	0.832
In active treatment, No. (%)	56 (62.2%)	28 (87.5%)	28 (48.3%)	**<0.001**
Prior therapy, No. (%)				overall *p* value 0.706
None	6 (6.7%)	1 (3.1%)	5 (8.6%)	
1st line	42 (46.7%)	14 (43.8%)	28 (48.3%)	
2nd line	25 (27.8%)	10 (31.3%)	15 (25.9%)	
≥3rd line	17 (18.9%)	7 (21.9%)	10 (17.2%)	
HSCT, No. (%) ^§^				overall *p* value **< 0.001**
>1 year prior	10 (11.1%)	1 (3.1%)	9 (15.5%)	**0.012**
≤1 year prior	19 (21.1%)	1 (3.1%)	18 (31%)	0.071
Anti-CD20 mAb therapy six months prior, No. (%)	23 (25.6%)	22 (68.8%)	1 (1.7%)	**<0.001**
Corticosteroid therapy six months prior, No. (%)	17 (18.9%)	6 (18.8%)	11 (19%)	0.980
Prednisone equivalent dose (mg), median (IQR)	133.3 (80–133.3)	120 (80–120)	133.3 (96.7–133.3)	0.173
Prednisone equivalent dose > 120 mg, No. (%)	10 (11.1%)	2 (6.3%)	8 (13.8%)	0.485

Abbreviations: *HSCT*, hematopoietic stem cell transplant; *mAb,* monoclonal antibody. °: aplastic anemia (*n* = 2), idiopathic thrombocytopenic purpura (*n* = 1); *: 7 missing data; ^+^: 26 missing data; ^§^: either Allo (*n* = 19), Auto (*n* = 9) or CAR-T (*n* = 1). Bold print: *p* value < 0.05.

**Table 2:** Significant predictors of no humoral response in patients who received two vaccine doses were determined at an α level of 0.05.

Variable	Univariable		Multivariable	
	RR [95% CI]	*p* Value	RR [95% CI]	*p* Value
Age > 67 years	2.44 [1.51–3.92]	< 0.001	2.57 [1.45–4.57]	0.001
Non-Hodgkin’s lymphoma	3.12 [1.86–5.21]	< 0.001		
Active treatment	1.68 [1.26–2.26]	< 0.001		
HSCT °	0.14 [0.04–0.55]	0.005		
HSCT > 1 year prior	0.12 [0.02–0.83]	0.031		
Anti-CD20 mAb therapy six months prior	6.41 [3.59–11.43]	0.002	4.88 [2.58–9.27]	< 0.001

Abbreviations: *HSCT*, hematopoietic stem cell transplant; *mAb*, monoclonal antibody. °: either Allo, Auto, or CAR-T.

## Data Availability

The data presented in this study is available upon request from the corresponding author. The data is not publicly available due to privacy restrictions.
